# Immune Responses to West Nile Virus Infection in the Central Nervous System

**DOI:** 10.3390/v4123812

**Published:** 2012-12-17

**Authors:** Hyelim Cho, Michael S. Diamond

**Affiliations:** 1 Departments of Molecular Microbiology, Washington University School of Medicine, St. Louis, Missouri 63110, USA; E-Mail: hyelimcho@wustl.edu; 2 Departments of Medicine, Washington University School of Medicine, St. Louis, Missouri 63110, USA; 3 Pathology and Immunology, Washington University School of Medicine, St. Louis, Missouri 63110, USA

**Keywords:** flavivirus, innate immunity, adaptive immunity, pathogenesis, immunopathogenesis, neuron, brain

## Abstract

West Nile virus (WNV) continues to cause outbreaks of severe neuroinvasive disease in humans and other vertebrate animals in the United States, Europe, and other regions of the world. This review discusses our understanding of the interactions between virus and host that occur in the central nervous system (CNS), the outcome of which can be protection, viral pathogenesis, or immunopathogenesis. We will focus on defining the current state of knowledge of WNV entry, tropism, and host immune response in the CNS, all of which affect the balance between injury and successful clearance.

## 1. Introduction

West Nile virus (WNV) is a mosquito borne, neurotropic, positive-stranded, enveloped RNA virus in the *Flaviviridae* family. WNV is related genetically to other viruses that cause severe visceral and central nervous system (CNS) diseases in humans including dengue (DENV), yellow fever (YFV), Japanese encephalitis (JEV), and tick-borne encephalitis (TBEV) viruses. WNV is maintained in an enzootic cycle between mosquitoes and birds, but also infects and causes disease in vertebrate animals including horses and humans. WNV is transmitted primarily by *Culex* species mosquitoes and the virus amplifies in bird reservoirs, with humans and horses largely considered as dead-end hosts [1]. Although human cases occur primarily after mosquito inoculation, infection after blood transfusion, organ transplantation, and intrauterine transmission has been reported [1]. At present, there are no vaccines or therapeutic agents approved for humans against WNV. 

WNV was first isolated in 1937 in Uganda from a woman with an undiagnosed febrile illness [2], and historically, has caused outbreaks of a relatively mild febrile illness in regions of Africa, the Middle East, Asia, and Australia [3]. In the 1990’s, the epidemiology of infection changed. New outbreaks in Eastern Europe were associated with higher rates of severe neurological disease [4]. In 1999, WNV entered North America, and caused seven human fatalities in the New York City area as well as a large number of avian and equine deaths. Since then, it has spread to all 48 of the lower continental United States as well as to parts of Canada, Mexico, the Caribbean, and South America. While the majority of human infections are asymptomatic, WNV can cause a severe febrile illness and neuroinvasive syndrome characterized by meningitis, encephalitis, and/or acute flaccid paralysis [5,6,7]. Persistent movement disorders, cognitive dysfunction, and long-term disability all occur after West Nile neuroinvasive disease. West Nile poliomyelitis-like disease results in limb weakness or paralysis. Patients show markedly decreased motor responses in the paretic limbs, preserved sensory responses, and widespread asymmetric muscle denervation without evidence of demyelination or myopathy [8]. Thus, the neurological and functional disability associated with WNV infection represents a considerable source of morbidity in surviving patients long after the acute illness [9,10,11,12,13]. In the United States alone between 1999 and 2012, ~36,000 cases and ~1,500 deaths have been confirmed. 

The risk of severe WNV infection in humans is greatest in the elderly and immunocompromised [14,15]. Two studies have estimated a 20-fold increased risk of neuroinvasive disease and death in those over 50 years of age [14,16]. Beyond age, a limited number of host genetic factors have been linked with susceptibility to WNV infection. A deficiency of the chemokine receptor CCR5 increases the risk of symptomatic WNV infection, as a higher incidence (4.2%) of loss-of-function CCR5Δ32 homozygotes was observed in symptomatic WNV infection cohorts compared to that in the general population (1.0%) [17]. A nonsense mutation in the gene encoding 2'-5'-oligoadenylate synthetase/L1 (OAS) isoform is associated with WNV susceptibility in laboratory mice [18]. Correspondingly, a hypomorphic allele of the human ortholog *OAS1* is associated with both symptomatic and asymptomatic WNV infection [19]. Finally, an association of single nucleotide polymorphisms (SNP) between symptomatic and asymptomatic WNV infections and *IRF3* and *Mx1* innate immune response and effector genes has been reported [20]; thus, genetic variation in the interferon (IFN) response pathway appears to correlate with the risk of symptomatic WNV infection in humans. In this review, we will summarize our understanding of the host-virus interface in the CNS and how this determines WNV disease pathogenesis and clinical outcome.

## 2. Virology and Pathogenesis

Although cellular receptors have not yet been identified definitively, studies suggest that WNV enters cells by endocytosis and fusion with the early endosome [21,22]. Following fusion between the viral and endosomal membranes, the nucleocapsid is released into the cytoplasm and 11 kilobase viral genomic RNA associates with endoplasmic reticulum (ER) membranes. The single open reading frame is translated into a polyprotein and enzymatically processed into three structural proteins (capsid (C), pre-membrane (prM)/membrane (M), and envelope (E)) and seven non-structural proteins (NS1, NS2A, NS2B, NS3, NS4A, NS4B, and NS5). Negative strand viral RNA then is synthesized and serves as a template for positive strand RNA synthesis [23]. Positive strand RNA is packaged in progeny virions, which bud into the ER to form enveloped immature virions. A maturation step, cleavage of the prM protein to the membrane M protein, occurs in the trans Golgi network by furin-like proteases [24,25,26] and results in a reorganization of E proteins on the virus surface into a homodimeric array [27]; these virions are secreted into the extracellular space by exocytosis.

Following mosquito inoculation into the skin, it is believed that WNV replicates within epidermal keratinocytes and Langerhans cells [28,29]. Migratory Langerhans dendritic cells enter afferent lymphatics and travel to draining lymph nodes [28]. Here, infection and the risk of dissemination are countered by the rapid development of an early immune response including type I and II IFN production and the effector functions of innate immune cells (γδ cells, NK cells, neutrophils, macrophages, and IgM-secreting B cells) [30,31,32,33,34]. Virus produced in the lymph node can enter circulation via the efferent lymphatic system and thoracic duct, and viremia allows spread to secondary lymphoid and visceral organs including the spleen and kidney [35,36]. In peripheral tissues, infection is restricted by innate and adaptive immune responses including serum IgM [37], IFN-α/β [38], IFN-γ [32,39], cytolytic CD8^+^ T cells [39,40,41], and cell-intrinsic IRF-3-dependent [30,42] antiviral responses. 

## 3. WNV-Induced Pathology in the CNS

WNV causes encephalitis in several vertebrate species by virtue of its ability to infect and injure neurons through direct (viral-induced) and indirect (immune response induced or bystander) mechanisms [43]. Pathologic observations in humans are limited by the small number of autopsy studies on individuals succumbing to WNV infection. In these few reports, gross macroscopic examination of the brain and spinal cord did not reveal any overt pathology [5]. Microscopic examination of the brain in humans and other animals reveals histological changes that are consistent with the clinical disease [5,36]. This includes neuronal cell death, activation of resident microglia and infiltrating macrophages, perivascular and parenchymal accumulation of CD4^+^ and CD8^+^ T cells and CD138^+^ plasma cells, and formation of microglial nodules. These lesions, which can be patchy in distribution, occur in the brainstem, cerebral cortex, the hippocampus, thalamus, and cerebellum [5]. Cellular infiltrates in the meninges also can be present. In some cases, destruction of vascular structures with focal hemorrhage occurs, suggestive of a vasculitis; this may be associated with local compromise of the blood-brain barrier (BBB) [44,45]. Immunohistochemical analysis confirms that WNV antigen is present primarily in neurons from multiple regions of the brain, although other cells (e.g., CD11b^+^ myeloid cells and possibly astrocytes) may be infected but to lesser degrees [46,47]. In the spinal cord, an intense inflammatory infiltrate around large and small blood vessels is observed with large numbers of microglia in the ventral horn. Anterior horn motor neurons are targeted by WNV [8,48], and studies suggest that axonal transport from peripheral neurons can mediate WNV entry into the spinal cord and induce acute flaccid paralysis [49]. Studies in hamsters reveal that limb paralysis and tremors are directly associated with infection and injury of anterior horn motor neurons in the lumbar section of the spinal cord [50]. 

## 4. Neuroinvasion

**Figure 1:**
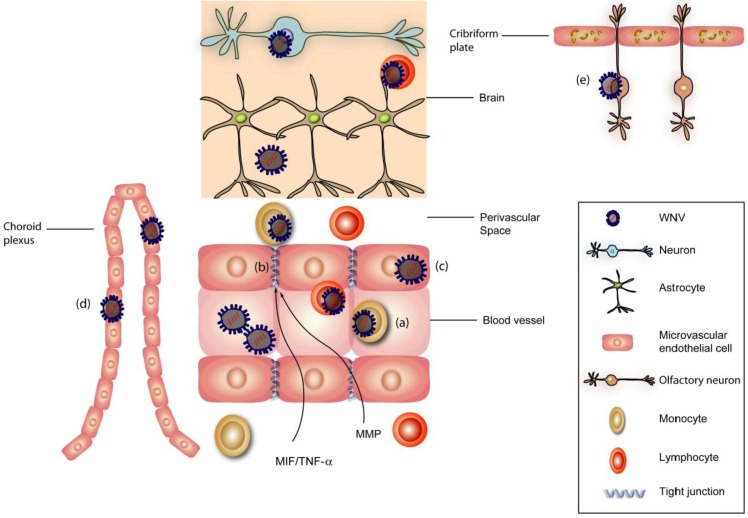
**Mechanism of neuroinvasion of West Nile virus (WNV).** WNV may enter the central nervous system (CNS) via multiple mechanisms including axonal retrograde transport along peripheral neurons into the spinal cord or hematogenous transport across the blood-brain barrier (BBB). Spinal cord entry is believed to result in interneuron spread to motor neuron cell bodies within the anterior horn of the spinal cord and lead to flaccid paralysis. The possible routes of virus entry across the BBB include (**a**) “Trojan horse” model; intracellular transport within macrophages or neutrophils, (**b**) loss of integrity of the BBB; cytokine-mediated (TNF-α, MIF) or matrix metalloproteinases disruption of tight junctions and basement membranes; (**c**) direct infection of brain microvascular endothelial cells with basolateral spread of the virus; (**d**) infection of choroid plexus epithelial cells; or (**e**) direct infection of olfactory neurons adjacent to the cribriform plate.

To establish infection in neurons of the brain, WNV first must cross the BBB (Figure 1). The BBB is composed of endothelial cells, astrocyte foot processes, and pericytes (PCs) and impedes the entry of macromolecules and pathogens from the blood into the brain. The tight junctions between endothelial cells form a diffusion barrier and pose obstacles for pathogens to enter the brain and to infect vulnerable and largely non-renewable neurons [51]. The mechanism by which WNV and other encephalitic flaviviruses cross the BBB remains uncertain. Crossing of the BBB likely occurs through a hematogenous route, as high levels of viremia correlate with greater and more rapid WNV entry into the CNS [52,53]. Intravascular levels of pro-inflammatory cytokines, which are produced during peripheral immune responses, also may modulate WNV entry into the CNS. WNV infection in peripheral tissues induces Toll-like receptor (TLR)-3-mediated secretion of pro-inflammatory cytokines, including IL-6 and TNF-α [44]. Secreted TNF-α can modulate BBB permeability by altering endothelial cell tight junctions, which may allow WNV to cross the BBB and infect neurons [44,54,55]. Semaphorin 7A upregulation after WNV infection also is linked to increased TNF-α production. Mice lacking Semaphorin 7A showed reduced TNF-α levels in serum, less BBB permeability, and reduced viral entry into the brain. [56]. Activation of matrix metalloproteinases also may enhance the flux of WNV by degrading the extracellular matrix of the BBB [57]. In BBB model studies *in vitro*, treatment with inhibitors of matrix metalloproteinases prevented the disruption of tight junction integrity associated with WNV infection [58]. 

Beyond compromise of the BBB, in some cases, WNV may penetrate into the CNS through additional mechanisms. Peripheral neurons are susceptible to infection by WNV [59,60]; retrograde axonal transport can bring WNV into the CNS, where transneuronal spread can occur. In contrast to some viruses (e.g., rabies [61]), neuron-to-neuron spread of WNV requires axonal release of viral particles [49]. Other possible entry mechanisms for WNV include (i) infection or passive transport through choroid plexus epithelial cells [62], (ii) a “Trojan horse” mechanism in which the virus is transported by infected immune cells (e.g., neutrophils [34] or CD4^+^ or CD8^+^ T cells [63]) that cross the BBB [64], (iii) infection of olfactory neurons and rostral spread from the olfactory bulb [65], or (iv) direct infection of brain microvascular endothelial cells [66]. The precise mechanism of WNV entry into the CNS in humans requires further study, and may differ depending on the route of infection and the pathogenicity of the WNV strain [67].

## 5. Neuronal Injury

WNV infection of neurons can result in caspase 3-dependent apoptosis, which likely contributes to CNS dysfunction and pathogenesis of severe disease. While no significant difference in peripheral or CNS tissue viral burden was observed in WNV-infected *caspase 3*^−/− ^mice, these animals were more resistant to lethal WNV infection due to reduced neuronal cell death in the cerebral cortex, brain stem, and cerebellum [68]. Consistent with this, ectopic expression of WNV NS2B-NS3 non-structural proteins activates caspase 3 and induces apoptosis in neuroblastoma cell lines [69], and primary neurons and neuroblastoma cells undergo apoptosis after WNV infection [48,68,70]. Cellular stress pathways including cAMP response element-binding transcription factor homologous protein (CHOP)-dependent apoptotic pathway also likely contribute to WNV-induced neuronal damage [71]. WNV infection may trigger apoptosis by activating non-caspase proteases, such as calpains and cathepsins [72,73]. Finally, WNV infection can induce non-apoptotic pathways of cell death. Cell necrosis can occur, as characterized by extensive cell swelling and loss of membrane integrity likely due to the extensive budding of WNV progeny virions into the ER [74]. 

In addition to injury imposed directly by WNV infection, neurons may undergo cell death or injury due to bystander damage caused by cytotoxic factors released by neuronal and non-neuronal cells. Neurons that are dying secondary to viral infection or immune-targeted death may release inflammatory molecules (e.g., Cxcl10 L-1β, IL-6, IL-8, and TNF-α) [75,76] with potentially toxic effects on uninfected neurons resulting in irreversible neuronal loss and atrophy. Analogously, glial cells, which are not primary targets of direct WNV infection, can become activated and release excitotoxic amino acids (e.g., glutamic and aspartic acids) and pro-inflammatory cytokines that contribute to the pathogenesis of neurological diseases by virus infections [77,78]. For example, TNF-α and IL1-β released by activated glial cells have direct roles in promoting bystander damage to neurons [79]. Elevated reactive oxygen species secreted by infected or activated microglial cells also may result in oxidative damage to neurons [80].

## 6. CNS Immune Responses to WNV

Upon entry in the CNS, WNV spreads rapidly between different subtypes of neurons in distinct regions [81]. As neurons are largely non-renewable, controlled immune responses must limit spread and eliminate virus while minimizing neuronal damage [82]. A delay or absence of such responses in genetically deficient mice or immunosuppressed humans results in rapid dissemination, neuronal injury, with an increased risk of mortality. Recent work in animal models has shown that both innate and cellular immune response in the CNS orchestrate control of WNV spread, which ultimately limits the number of neurons targeted for infection or the amount of virus any given infected neurons will produce. 

### 6.1. CNS Innate Immunity

Nucleic acid intermediates of RNA virus replication are recognized by pathogen recognition receptors (PRR) such as TLR and RIG-I like receptors (RLR), which promote an antiviral state by activating IRF-3 and IRF-7-mediated transcriptional programs and type I IFN responses. The importance of these pathways for controlling WNV infection is highlighted by studies in mice that are genetically deficient for key components in this pathway: Type I IFN receptor-knockout mice (*Ifnar*^−/−^), *Ifnb*^−/−^, *Mavs*^−/−^, *Tlr3*^−/−^, *Tlr7*^−/−^, and *Myd88*^−/− ^mice all show enhanced viral replication in the CNS and mortality after WNV infection [38,47,83,84,85]. *Irf3*^−/−^ neurons showed reduced induction of antiviral defense genes including *Rig-I*, *Mda5*, and *Ifit1*, as well as blunted IFNα/β production [42]. In *Irf7*^−/−^ neurons, IFN-α production was blunted, which resulted in increased WNV infection [86]. Together, these studies suggest that IRF-3 and IRF-7-dependent transcriptional programs are crucial for protective IFN response in neurons. Stat1-dependent signaling pathways in part, determine the susceptibility of specific neuronal subtypes to WNV infection in the brain. IFN-α/β and Stat1-dependent transcription of IFN-stimulated genes (ISGs) inhibited WNV replication in neurons *in vitro* and *in vivo*. Rsad2 (also known as viperin), PKR, and RNase L are induced in neurons of the CNS and restrict WNV infection *in vivo* [87,88].

### 6.2. Inflammatory Responses

**Figure 2:**
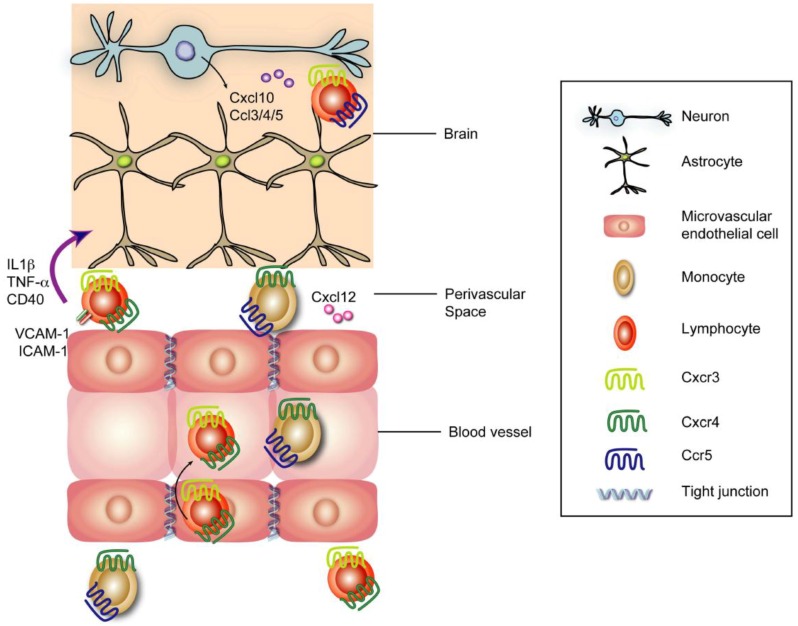
**Leukocyte trafficking into the CNS after WNV.** Upon WNV infection of neurons, virus-mediated upregulation of Cxcl10 recruits virus-specific CD8^+^ T cells via interactions with Cxcr3. Expression of Ccl3, Ccl4, and Ccl5 by other neuronal cells recruits Ccr5-expressing leukocytes. Monocytes and lymphocytes entering the perivascular spaces may be retained initially via Cxcr4 binding Cxcl12 [120]. Leukocyte egress from perivascular spaces requires IL-1β, TNF-α, and CD40 interactions, which likely upregulates adhesion molecules including ICAM-1 and VCAM-1 [121,123,124].

Neurons in the CNS are immunologically active and initiate inflammatory responses by producing chemokines that recruit immune cells (Figure 2). Infection of neurons by WNV induces expression of the T cell chemoattractant Cxcl10, which promotes trafficking of WNV-specific CD8^+^ T cells via binding to its cognate receptor Cxcr3 [89,90]. Enhanced expression of Ccl3 (MIP-1α), Ccl4 (MIP-1β), Ccl5 (RANTES) by WNV infection leads to Ccr5-dependent trafficking of CD4^+^ and CD8^+^ T cells, NK cells, and macrophages. Deletion or truncation of Ccr5 in mice leads to enhanced viral burden and increased mortality [91,92], and appears to be associated with more severe disease in humans [17]. Trafficking of monocytes into the brain, as precursors of macrophages and possibly microglia, can contribute to CNS injury [93] or survival after WNV infection [94], depending on the virulence of the infecting WNV strain. In mice, deletion of Ccr2, a chemokine receptor on inflammatory monocytes, leads to increased mortality after infection by virulent North American WNV strains, and this is associated with reduced monocyte accumulation in the brain [94]. Study with *Il22*^−/−^ mice demonstrate that reduced levels of Cxcr2, a chemokine receptor mediating neutrophil migration, correlate with decreased viral loads in the CNS [95], suggesting that entry of WNV-infected neutrophils may contribute to pathogenesis. In *Tlr7^−/−^* mice, CD45^+^ leukocytes and CD11b^+^ macrophages failed to home to WNV-infected neurons due to blunted IL-23 responses, suggesting *Tlr7* reduces WNV infection in part, by enhancing IL-23-dependent immune cell infiltration and homing into the brain [85]. 

### 6.3. Cellular Immunity

Studies in mice suggest that T cell-mediated immunity is an essential aspect of immune mediated protection from virulent strains of WNV. The lack of a functional CD4^+^ and CD8^+^ T cell response results in inefficient clearance of WNV infection from neurons of the brain [39,40,96]. Nonetheless, an over-exuberant CD8^+^ T cell-mediated response can lead to injury and or death of infected or uninfected neurons. In mice, within a few days of CNS infection, inflammatory cytokines and chemokines produced by resident cells of the CNS attract antigen-specific CD8^+ ^T cells into the CNS [40,89]. In addition, CD40-CD40L and TNFα-TNFα-receptor interactions promote CD8^+^ T cell migration across brain microvascular endothelial cells, likely by increasing expression of adhesion molecules and modulating the integrity of tight junctions [97,98].

**Figure 3:**
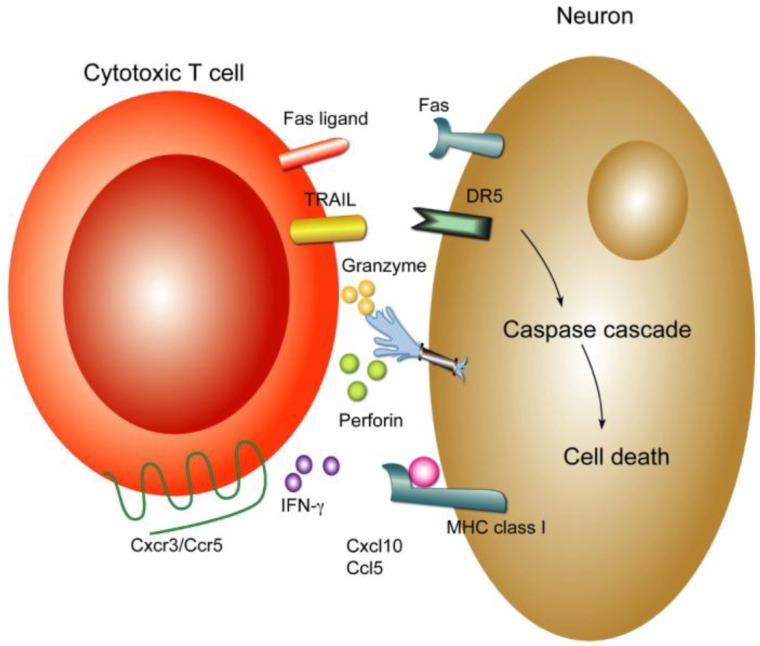
**Mechanisms of CD8^+^ T cell clearance in the CNS.** CD8^+^ T cells control WNV infection in the CNS through multiple mechanisms. Infected neurons upregulate surface expression of MHC class I molecules. Antigen-specific CD8^+^ T cells recognize infected neurons via class I MHC and processed viral peptides and trigger cell death of target cells through perforin, Fas-Fas ligand, or TRAIL-dependent pathways. Perforin-mediated control of infected neurons occurs through the granzyme-dependent granule exocytosis pathway, which results in apoptosis of infected neuron. Interactions between Fas on infected neurons and FasL on CD8^+^ T cells leads to programmed cell death of neurons through caspase-dependent pathways. CD8^+^ T cells also utilize TRAIL to restrict WNV infection in neurons. TRAIL binds to DR5 on neurons, which can have a direct antiviral effect against flaviviruses [125] or result in targeted apoptosis. Activated CD8^+^ T cell also produce IFN-γ, which can induce genes with antiviral effect.

CD8^+^ T cells control WNV infection in the CNS via multiple mechanisms (Figure 3) including the production of antiviral cytokines (e.g., IFN-γ) or by triggering cell death of target cells through perforin, Fas-Fas ligand, or TRAIL-dependent pathways. Infected neurons up-regulate MHC class I molecules and thus, can be targeted by cytotoxic T cells [99]. *Perforin*^−/−^ mice showed higher viral burden in CNS and increased mortality after WNV infection [39], as well as a failure to clear WNV resulting in persistent CNS infection. Perforin-mediated control of infected neurons occurs through the granzyme-dependent granule exocytosis pathway, which results in apoptosis of infected neurons *in vitro* and *in vivo* [100,101,102]. Fas ligand (FasL) deficient mice also showed increased susceptibility to lethal WNV infection [103]. Interactions between Fas on infected neurons and FasL on CD8^+^ T cells leads to programmed cell death of neurons through the activation of a death domain and a caspase apoptosis cascade [102,104,105]. CD8^+^ T cells also use tumor necrosis factor-related apoptosis-inducing ligand (TRAIL; also known as CD253) to restrict WNV pathogenesis by controlling infection in neurons. TRAIL binding to the death receptor DR5 on neurons activates a caspase-dependent apoptosis cascade [41]. Consistent with results establishing a protective effect of effector CD8^+^ T cells in mice, humans with impaired T cell immunity have a greater risk of CNS infection with WNV [106]. 

Although T cell responses are important for viral clearance, they can cause irrevocable damage to the host. Under certain conditions, infection of mice lacking CD8^+^ T cells with an attenuated lineage 2 WNV (Sarafend) strain resulted in decreased morbidity and mortality compared to wild type mice [107]. Consistent with this, depletion of CD8^+^ T cells in mice infected with an attenuated genetic variant of a North American WNV strain resulted in prolonged survival [108]. Thus, depending on the virological and immunological context, CD8^+^ T cells either can protect against or contribute to WNV neurological disease. 

## 7. Viral Persistence in the CNS

Although still controversial, persistent WNV infection and inflammation in the CNS of vertebrate animals has been reported in mice, monkeys, and hamsters [109,110,111,112,113]. These results are consistent with earlier studies in animals and humans showing flavivirus persistence after infection with Saint Louis encephalitis, tick-borne encephalitis, and louping ill viruses [114,115,116,117]. In monkeys, the duration of WNV persistence was at least 5.5 months, with infectious virus isolated from the cerebellum and cerebral subcortical ganglia. Virus recovered more than two months after initial infection from these monkeys retained neurovirulence [111]. In hamsters, WNV persistence has been described up to 86 days after initial infection, and this was associated with long term neurological sequelae [112,113]. In mice, infectious WNV was detected in the brains up to 4 months in 12% of mice and viral RNA persisted up to 6 months after infection [110]. Consistent with this, virus-specific B and T cell immune responses persisted in the brains of mice for at least 4 months after infection [109]. Although viral persistence in the CNS has not been documented in humans, chronic WNV infection in the kidney has been reported in some patient cohorts [118,119].

## 8. Summary and Future Perspectives

WNV continues to spread and cause neurological disease and thus, remains a public health concern in the United States and other countries. Research into the viral and host factors that determine the pathogenesis and outcome of WNV infection is crucial for development of new therapeutic and vaccines strategies. A more complete understanding of the mechanisms of immunopathogenesis in the CNS could facilitate the development tailored anti-inflammatory agents that minimize neuronal damage without preventing clearance. As examples, treatment with the Cxcr4 antagonist AMD3100 enhanced CD8^+^ T cell trafficking into the parenchyma of CNS and improved survival after WNV encephalitis [120], whereas blockade of migration of nitric oxide-producing inflammatory macrophage using anti-very late antigen (VLA)-4 integrin antibody prolonged survival after WNV encephalitis [121]. Combining such types of immunomodulatory agents with small molecule or antibody-based antiviral molecules [122] that target viral replication or tropism might be a way to maximize viral clearance and minimize neuropathogenesis after WNV infection.
